# Traits associated with innate and adaptive immunity in pigs: heritability and associations with performance under different health status conditions

**DOI:** 10.1186/1297-9686-41-54

**Published:** 2009-12-30

**Authors:** Mary Clapperton, Abigail B Diack, Oswald Matika, Elizabeth J Glass, Christy D Gladney, Martha A Mellencamp, Annabelle Hoste, Stephen C Bishop

**Affiliations:** 1 The Roslin Institute and Royal Dick School of Veterinary Studies, University of Edinburgh, Roslin, Midlothian, EH25 9PS, UK; 2 Faculty of Veterinary Medicine, University of Glasgow, G61 IQH, UK; 3 Genus, De Forest, WI 53532, USA; 4 Ralco Nutrition, Inc., 1600 Hahn Road, Marshall, MN 56258, USA; 5 JSR Genetics Ltd, Driffield, East Yorkshire, Y025 9ED, UK

## Abstract

There is a need for genetic markers or biomarkers that can predict resistance towards a wide range of infectious diseases, especially within a health environment typical of commercial farms. Such markers also need to be heritable under these conditions and ideally correlate with commercial performance traits. In this study, we estimated the heritabilities of a wide range of immune traits, as potential biomarkers, and measured their relationship with performance within both specific pathogen-free (SPF) and non-SPF environments. Immune traits were measured in 674 SPF pigs and 606 non-SPF pigs, which were subsets of the populations for which we had performance measurements (average daily gain), viz. 1549 SPF pigs and 1093 non-SPF pigs. Immune traits measured included total and differential white blood cell counts, peripheral blood mononuclear leucocyte (PBML) subsets (CD4^+ ^cells, total CD8α^+ ^cells, classical CD8αβ^+ ^cells, CD11R1^+ ^cells (CD8α^+ ^and CD8α^-^), B cells, monocytes and CD16^+ ^cells) and acute phase proteins (alpha-_1 _acid glycoprotein (AGP), haptoglobin, C-reactive protein (CRP) and transthyretin). Nearly all traits tested were heritable regardless of health status, although the heritability estimate for average daily gain was lower under non-SPF conditions. There were also negative genetic correlations between performance and the following immune traits: CD11R1^+ ^cells, monocytes and the acute phase protein AGP. The strength of the association between performance and AGP was not affected by health status. However, negative genetic correlations were only apparent between performance and monocytes under SPF conditions and between performance and CD11R1^+ ^cells under non-SPF conditions. Although we cannot infer causality in these relationships, these results suggest a role for using some immune traits, particularly CD11R1^+ ^cells or AGP concentrations, as predictors of pig performance under the lower health status conditions associated with commercial farms.

## Background

The control of infection represents a major challenge to the pig industry. Over the last decade, this challenge has become greater due to the spread of viral infections such as PMWS (post-weaning multi-systemic wasting syndrome), PRRS (porcine reproductive and respiratory syndrome) and enzootic pneumonia. In addition to the impact of these infections or diseases upon pig morbidity and mortality, they can also affect pig health by increasing susceptibility to secondary bacterial infections [1-3].

Since antibiotics and bio-security control measures can only partially control infection, and effective vaccines are not always available, it would be advantageous to find a method of selecting pigs with increased resistance to a wide range of infectious diseases or an increased ability to maintain high performance levels in the face of disease pressure. In pig breeding companies, pigs are generally selected for improved performance within the high health status environment of a nucleus farm, but often their progeny are reared within a lower health status environment and, as a result, their performance may be compromised. Hence there is a need to find a way of selecting boars that can produce progeny with an increased resistance to a wide range of infectious diseases so that they are able to perform well under a range of health conditions.

In pig production systems it is difficult to select animals directly for disease resistance since the major challenges often differ in different environments and most husbandry practices attempt to minimise exposure to infection. Therefore, an alternative approach is needed. One such approach would be to use measures of innate and adaptive immunity which are heritable and associated with parameters related to health and/or performance. In order to predict progeny that will perform equally well in a range of environments, these immune markers would have to be heritable regardless of health status.

Previously, we have shown peripheral blood mononuclear leucocyte (PBML) subsets to be heritable [4]. Further, CD11R1^+ ^cells, a subset consisting of natural killer (NK) cells and NK T cells, [5,6] were also genetically negatively correlated with performance [4]. It may be hypothesized that this type of association reflects an underlying response to infection, and this result can be explored by comparing the genetic relationship between CD11R1^+ ^cells and performance under both high and lower health status environments. Significant genetic relationships with performance under lower health status environments would suggest that they can be used as biomarkers for health or performance in such environments. We still need to satisfactorily quantify the effect of health status on the properties of these immune traits, particularly their heritabilities and correlations with performance.

Added insight into the utility of measuring the PBML subsets may also be gained by refining their definitions. For example, in our previous study [4], we did not account for the presence of the different CD8α^+ ^subsets that are unique to pig PBML. In addition to classical CD8αβ^+ ^cells, these subsets include CD4^+^CD8α^+ ^cells, CD8α^+ ^γδ^+ ^T cells and CD8α^+ ^NK cells [7]. In particular, CD4^+ ^CD8α^+ ^cells have been suggested to be memory CD4^+ ^helper cells [8,9] and hence, an important component of the adaptive immune response. It is also possible to distinguish between CD8αβ^+ ^cells and CD8αα^+ ^subsets on the basis of CD8α expression since CD8αβ^+ ^cells express higher levels of CD8 antigen compared to CD8αα^+ ^cells [7]. Further PBML subsets of importance that we can define include CD14^+ ^and CD16^+ ^cells. Within pig PBML, CD16 is expressed on NK cells and monocytes [6,10,11] whilst, in pigs, CD14 is a marker of monocyte differentiation [12]. Lastly, CD11R1^+ ^cells may be sub-divided into CD8α^+ ^and CD8α^- ^subsets since these cell subsets differ according to cell size, complexity and phenotype [13] (Clapperton, unpublished observations).

In addition to PBML subsets, we have also reported that acute phase proteins (APP) have a negative phenotypic correlation with daily weight gain and food efficiency [14]. One possible interpretation of this effect is that sub-clinical infection simultaneously leads to both decreased weight gain and food efficiency and increased APP levels. This study [14] also found pig line differences in the levels of the acute phase protein, alpha-_1 _acid glycoprotein, which suggested that APP levels may also be under genetic control. APP such as haptoglobin, transthyretin, alpha-_1 _acid glycoprotein (AGP) and C-reactive protein (CRP) have also been shown to act as potential indicators of animal and farm health status [15-19]. Therefore, these APP may be valuable as genetic predictors of pig health, the hypothesis being that low APP values predict increased performance as a result of lower levels of infection in selected offspring.

This paper provides a comprehensive analysis of PBML and APP measurements, and their genetic relationships with performance, extending our previous results [4]. In particular, we provide the first estimates of heritability for all APP that were measured and the newly defined PBML. Importantly, by substantially increasing the size of our dataset we can also compare the heritabilities of a large range of immune traits, and their associations with performance, between high and lower health status environments. These results should indicate the extent to which host genotype influences both the basal levels of these traits and their levels in response to exposure to pathogens.

## Methods

### Populations studied and performance trait measurements

Measurements were performed on pigs sampled from seven farms labelled A to G. Details of numbers of pigs tested per farm along with the number of sires and full-sib families (i.e. litters) are shown in Table 1. All pigs tested were apparently healthy with no clinical signs of infection. Farm G represented the Roslin Institute farm whilst farms A to F represented farms from one of the three pig breeding companies (sources 1-3) who contributed animals to the study and are cited in the acknowledgements.

**Table 1:** Numbers of pigs tested, sires, lines and families per farm^1^

Source	1			2				3			4
Farm	A			B		D	E	C	F		G
Health status	SPF			SPF		Non-SPF		SPF	Non-SPF		Non-SPF
Generation	G1	G2	GX	G1	G2	G2	GX	G1	G2	GX	GX
No. pigs:											
Performance	47	684	373	92	259	148	300	94	398	72	175
Immune traits	47	0	373	92	68	59	300	94	0	72	175
APP	0	0	373	0	0	0	300	0	0	72	175
No. groups:											
Sires	21	4^2^	27	23	5 ^2,3^	5 ^2,3^	17	10	5^2^	11	55
Full-sib families	33	155	200	53	53	53	121	19	53	18	72
Genetic lines	8	4	2	1	1	1	1	1	1	8	13
Breed	LW	LW	LW	LW	LW	LW	LW	LW	LW	LR	LW

^1 ^For each source, G2 progeny were derived from G1 boars. All pigs tested for immune traits also had performance measurements. 2642 pigs were performance tested, 1280 pigs were tested for immune traits and 920 pigs were tested for APP. ^2 ^All sires were measured when they were growing pigs. ^3^These were the same sires.

In all cases, sows were reared on the same farm as the offspring. After birth, the offspring remained with their dams until age four weeks, whereupon they were weaned and transferred to flat deck pens and kept in groups of 28-20. At start of test (ca. 10-13 weeks of age), animals were split into groups (less than 20 animals) until end of test, except for Farm G where animals were housed in individual pens. All animals were housed in straw bed pens. There was variation between farms with respect to the type of buildings used and ventilation.

Farms A, B and C were classified as specific pathogen-free (SPF), i.e. free of all major swine pathogens whilst farms D, E, F and G were classified as non-SPF. Farms D-G were free of all major swine pathogens, as determined by clinical examination and serology tests, except for the following: Farm D was tested positive for enzootic pneumonia (*Mycoplasma hyopneumoniae*) on the basis of serology and clinical signs, and Farms E and F were positive for porcine multi-wasting syndrome (PMWS) on the basis of clinical signs. Farm G was positive for *Pasteurella multocida, Actinobacillus pleuropneumoniae, Leptospira bratislava *and also, *Salmonella typhimurium *phage type 104 was detected in faecal samples from this farm.

A detailed breakdown of the collected data is given in Table 1. Data from sources 1-3 were split into three generations, G1, G2 and GX. A small number of sires were selected from G1 by the breeding companies on performance attributes and used to produce progeny (G2) using unrelated dams on the same farm. Immune traits were measured in all G1 animals and a sample of G2 animals chosen at random, whilst performance was measured in all G1 and G2 animals. The G2 pigs located on both the SPF and non-SPF farms at source 2 were progeny of the same sires. Generation GX animals comprised populations from the same breeding companies/lines as G1 or G2; however their data (immune measures and performance) were collected three or more years later, and genetic relationships between GX and G1 or G2 were sparse and not included in the analyses. In general, different pig breed-lines were used on different farms, except on farms B and E, and C and F where common lines were used. Also Farm F comprised Landrace as well as Large White pigs. Approximately equal numbers of males and females were measured.

Animals were blood sampled at end of test (ca. 90 kg) by collecting blood via the external jugular vein into a tube containing EDTA or acid citrate dextrose anti-coagulant for leucocyte subset measurements and a tube containing lithium heparin for acute phase protein measurements. Sampling was staggered so that pigs were tested in weekly groups of 20 to 30 pigs in all farms except Farm G. Sampling for each farm was completed within a period of 3 to 8 weeks except for Farm G where sampling was performed on groups of 6-8 pigs over a twelve month period. The liveweights obtained at the start of test (ca. 30 kg) and at the end of test were retained in this dataset and used to calculate average daily gain for both blood sampled animals and their non-sampled littermates.

All procedures performed on the animals tested in this study were approved by the relevant government authorities responsible for animal welfare.

### Immune measurements

Leucocyte subset measurements and the storage of plasma samples for acute phase protein measurements occurred within 72 h after blood collection. During this time, blood was stored at room temperature. Total and differential white blood cell counts (WBC) were measured as described previously [13]. The proportions of different peripheral blood mononuclear leucocyte (PBML) subsets were measured as described previously [13], using flow cytometry and primary monoclonal antibodies that recognized cell surface markers for CD4, CD8α, gamma delta (γδ) T cell receptor, immunoglobulin light chain (B cell marker), CD11R1 (NK cell marker) and SIRPα (monocyte marker). In addition, we added markers for CD14^+ ^monocytes (clone MIL-2; [20]) and CD16^+ ^cells (clone G7; [10,11]). Our measurements also incorporated the following CD8α subsets - CD4^+ ^CD8α^+ ^cells and CD4^+^CD8α^- ^cells, CD11R1^+^CD8α^+ ^and CD11R1^+^CD8α^- ^cells.

CD8α^+ ^cells sub-divide into two clearly distinct subsets based upon the intensity of staining for CD8α^+^, into 'bright' and 'dim' populations as previously described [7]. CD8α^+ ^'bright' cells were CD8α^+ ^cells with high intensity of expression for CD8α, and CD8α^+ ^'dim' cells were CD8α^+ ^cells with low intensity of expression for CD8α. The antigen density for both the different CD8α^+ ^populations and for the CD8β^+ ^population was calculated using Qifikit beads (Dako Cytomation, Ely, Cambridgeshire) according to the manufacturer's instructions.

Plasma from a 5 mL blood sample was used for the measurement of the acute phase proteins, viz. AGP, haptoglobin, CRP and transthyretin. Plasma was collected from each blood sample after centrifugation at 1000 × g for 10 min and then decanted into a polypropylene tube and stored at -20°C. AGP was measured using a commercial kit based on a radial immuno-diffusion assay according to the manufacturer's instructions (The Metabolic Institute, Tokyo, Japan). Transthyretin and CRP were measured using an ELISA as described previously [21,22]. The concentration of haptoglobin was derived from its haemoglobin binding activity, as described by Eckersall *et al*. (1999) [23].

### Data analysis

Traits selected for analysis were average daily gain and the following immune traits, total and differential WBC count, proportions of PBML subsets and APP levels. PBML subset proportions included CD8α^+ ^cells, CD11R1^+ ^cells (CD8α^+ ^and CD8α^- ^subsets), CD4^+ ^T cells (CD8α^+ ^and CD8α^- ^subsets), γδ^+ ^T cells, B cells, monocytes (SIRPα^+ ^cells), CD14^+ ^cells (monocyte subset) and CD16^+ ^cells (NK cells and monocytes) and APP included AGP, haptoglobin, CRP and transthyretin.

An initial analysis of the data was performed using GENSTAT [24] to determine significant fixed effects and to characterize the data. Since the distributions of most traits were skewed to the right, log transformations were required to normalise the data for these traits prior to analysis. The proportions of mononuclear and polymorphonuclear cells were instead square root transformed. Significant fixed effects for most traits included farm, generation and genetic line nested within farm, sex and age at blood sampling. For the non-SPF animals, disease status was confounded with farm, i.e. different farms had different diseases, and Farm F had Landrace as well as LW pigs.

Genetic parameters and their standard errors were estimated using the AS-REML package [25], fitting an animal model including all known pedigree relationships. Each trait was fitted against the fixed effects described above and the random effects fitted in all analyses were the residual term, the effect of pen plus the direct genetic effect. For one trait (transthyretin) a general maternal effect, which could contain both genetic and environmental (litter) effects, was also significant and fitted. Uni-variate and bi-variate analyses were performed for each trait described above using all available data for each trait from all animals shown in Table 1, i.e. including animals with performance data but no immune measurements as well as those with both sets of data. The uni-variate and bi-variate analyses for each trait were then repeated using data from either only SPF and non-SPF farms. Uni-variate analyses were performed for all traits, bi-variate analyses were targeted at specific hypotheses.

In order to test whether differences in heritability estimates between SPF and non-SPF conditions were significant, a t value was estimated as:

## Results

### Characteristics of data within specific pathogen-free (SPF) and non-SPF environments

Table 1 shows the details of the numbers of pigs tested along with the number of generations, genetic lines and pedigree details for each farm. Table 2 summarises the data for all immune and performance traits tested within each type of environment. Health status did not affect either the mean values or the variances for any of the traits tested with the exception of AGP, which was lower under non-SPF conditions than SPF conditions (p < 0.01). This difference could have been caused by differences in either health status or line, as these factors were confounded in the dataset.

**Table 2:** Summary of immune and performance traits for pigs from SPF and non-SPF farms^1^

Health status	SPF	Non-SPF
Number of pigs tested		
- immune traits	674	606
- performance traits	1549	1093
		
Measurement (units)	Mean (variance)	Mean (variance)
		
White blood cells	22.2 (16.9)	24.0 (9.4)
MNL%	70.2 (9.10)	71.2 (11.6)
PMNL%	29.9 (9.10)	28.9 (11.6)
		
PBML subsets:		
		
CD4^+^	17.8 (4.87)	18.0 (5.64)
CD8α^+^	28.8 (7.16)	27.4 (6.69)
CD4^+^CD8α^+^	7.51 (2.99)	7.29 (3.30)
CD4^+^CD8α^-^	10.2 (3.83)	11.3 (3.79)
CD8αβ^+^	14.2 (4.57)	11.8 (4.02)
CD11R1^+ ^total	14.0 (4.49)	14.1 (5.38)
CD11R1^+^CD8α^+^	5.57 (2.94)	4.48 (2.61)
CD11R1^+^CD8α^-^	8.37 (3.11)	8.43 (3.65)
γδ^+ ^T cells	29.2 (8.36)	33.2 (12.5)
B cells	12.4 (5.33)	14.5 (6.19)
Monocytes	10.6 (4.85)	9.86 (4.09)
CD14^+^	5.21 (2.48)	6.18 (3.45)
CD16^+^	18.0 (5.00)	18.4 (6.26)
		
APP, μg/ml:		
		
Haptoglobin	0.78 (0.62)	0.69 (0.65)
TTR	442.6 (170.0)	555.3 (141.7)
CRP	145.6 (164.6)	144.4 (133.0)
AGP	744.8 (278.7)	388.3 (165.3)
		
ADG, kg/d	0.86 (0.16)	0.85 (0.17)
		
age (d), at start-test	80.9 (9.4)	94.7 (10.1)
age (d), at end-test	146 (10.4)	151 (12.1)
		
Ag density:		
		
all CD8α^+ ^cells	34185 (22820)	28936 (6936)
CD8α^+ ^"dim" cells	15423 (9521)	13422 (3330)
CD8α^+ ^"bright" cells	66368 (42468)	61150 (14483)
all CD8β^+ ^cells	27046 (16257)	20692 (4932)

^1 ^White blood cells expressed as no. cells × 10^6^/mL, PBML sub-sets expressed as proportion of mononuclear leucocytes and antigen density expressed as the number of antibody binding sites per cell (see Materials and Methods).

### Effect of health status on trait heritabilities

Estimated heritabilities obtained using the entire dataset are shown in Table 3, for all measured traits. Overall, most of the traits tested were moderately to highly heritable and significantly different from zero (p < 0.05). In particular, classical cytotoxic CD8αβ^+ ^cells and the CD4^+ ^subsets were both highly heritable, with values ranging from 0.37 to 0.75. The expression of CD8α and CD8β antigens were also highly heritable, ranging from 0.73 to 0.93. However, for CD16^+ ^cells, the heritability was low and not significantly different from zero (h^2 ^(s.e.) 0.09 (0.08)). For acute phase protein transthyretin, there was a strong maternal effect which, if not fitted, resulted in an inflated heritability estimate (data not shown). For some traits, the variation contributed by the local environment, represented by the effect of pen, was also significant (Table 3).

**Table 3:** Estimates of direct heritability and pen^1 ^variance ratios for immune traits and average daily gain^2, 3^

Trait:	Direct h^2 ^(s.e.)	Pen variance/σ^2 ^p
White blood cells	0.28 (0.08)	NS
MNL	0.21 (0.09)	0.13 (0.04)*
PMNL	0.24 (0.10)	0.10 (0.04)*
		
PBML subsets:		
		
CD4^+^	0.69 (0.09)	0.05 (0.03)*
CD8α^+^	0.46 (0.10)	NS
CD4^+^CD8α^+^	0.37 (0.11)	NS
CD4^+^CD8α^-^	0.75 (0.13)	NS
CD8αβ^+^	0.45 (0.11)	NS
CD11R1^+ ^total	0.35 (0.09)	NS
CD11R1^+^CD8α^+^	0.38 (0.10)	NS
CD11R1^+^CD8α^-^	0.25 (0.09)	0.10 (0.05)*
γδ^+ ^T cell	0.39 (0.09)	NS
B cells	0.31 (0.09)	NS
Monocytes	0.28 (0.09)	NS
CD14^+^	0.20 (0.11)	NS
CD16^+^	0.09 (0.08)	0.08 (0.04)*
		
APP, μg/mL		
		
Haptoglobin	0.23 (0.09)	0.07 (0.04)*
TTR	0.21 (0.15)	0.25 (0.08)*
CRP	0.15 (0.08)	0.07 (0.04)*
AGP	0.48 (0.10)	0.08 (0.04)*
		
ADG, kg/d	0.25 (0.06)	NS
		
Ag density:		

all CD8α^+ ^cells	0.73 (0.17)	NS
CD8α^+ ^"dim" cells	0.93 (0.16)	NS
CD8α^+ ^"bright" cells	0.73 (0.16)	NS
all CD8β^+ ^cells	0.85 (0.15)	NS

^1 ^Pen was fitted as a random effect for all traits, but the pen variance is only presented when significant. For transthyretin the pen variance was not significant, and the maternal variance as a proportion of phenotypic variance is presented instead. Maternal effects were not significant and were not fitted for all other traits.

^2^Mean pig weight at time of measurement was 90 kg.

^3 ^White blood cells expressed as no. cells × 10^6^/mL, PBML sub-sets expressed as proportion of mononuclear leucocytes and antigen density expressed as the number of antibody binding sites per cell (see Materials and Methods).

* p < 0.05.

In Tables 4 and 5, the effects of health status on the direct heritability of immune and performance traits along with the genetic and residual variances are shown. The effect of pen was also fitted to all traits, and is shown in cases where it is significant. Because differences in estimated heritabilities may be due to changes in either the genetic or environmental variance, both of these variance components are also presented. There is a tendency for the transition from a high to a lower health status to be associated with a decrease in heritability, and this change tended to be more associated with a decrease in the genetic variance than an increase in the residual variance. The residual variance was often relatively stable particularly for the acute phase proteins. Notably, the proportions of CD11R1^+ ^cells and CD16^+ ^cells were moderately heritable under SPF conditions (h^2 ^(s.e.) 0.46 (0.12)) but lowly heritable under non-SPF conditions (h^2 ^(s.e.) 0.07 (0.08)), although for CD11R1^+ ^cells the local environmental effect, i.e. the pen variance, appeared to be high compared to the average pen variance for other traits. When the pen effect was not fitted, the estimated heritability for this measurement was 0.36 (0.14). When the variance for 'pen' was fixed to be the same as for SPF pigs, the estimated heritability (± se) for this measurement was 0.29 (0.14).

**Table 4:** Direct heritability (h^2^) estimates for total and differential WBC and PBML subsets for SPF and non-SPF pigs^1, 2,^

Farm	SPF				Non-SPF			
Trait	Direct h^2 ^(s.e.)	Pen effect (s.e.)	Genetic variance × 10^-1^	Residual variance × 10^-1^	Direct h^2 ^(s.e.)	Pen effect (s.e.)	Genetic variance × 10^-1^	Residual variance × 10^-1^
WBC	0.29 (0.13)	NS	0.32	0.72	0.28 (0.11)	NS	0.21	0.55
								
MNL%	0.09 (0.10)	0.16 (0.06)*	0.03	2.02	0.29 (0.13)	NS	0.96	2.10
PMNL%	0.09 (0.10)	0.17 (0.06)*	0.56	4.60	0.36 (0.14)	NS	3.00	5.20
								
PBML subsets:								
								
CD4^+^	0.78 (0.12)	NS	187	38.3	0.57 (0.14)	0.06 (0.04)*	147	94.1
CD8α^+^	0.35 (0.12)	NS	168	306	0.60 (0.16)	0.12 (0.05)*	215	141
CD4^+^CD8α^+^	0.34 (0.15)	NS	0.58	1.10	0.37 (0.17)	NS	0.46	0.79
CD4^+^CD8α^-^	0.82 (0.17)	NS	0.95	0.19	0.45 (0.21)	NS	0.35	0.43
CD8αβ^+^	0.48 (0.16)	NS	0.42	0.45	0.36 (0.17)	NS	0.32	0.56
CD11R1^+ ^total	0.46 (0.12)	NS	0.45	0.52	0.07 (0.08)	0.34 (0.08)*	0.09	0.78
CD11R1^+^CD8α^+^	0.46 (0.13)	NS	0.11	0.13	0.31 (0.16)	NS	0.73	1.60
CD11R1^+^CD8α^-^	0.27 (0.11)	0.13 (0.06)*	0.39	0.88	0.27 (0.14)	NS	0.30	0.70
γδ^+ ^T cells	0.46 (0.12)	0.05 (0.04)	233	279	0.30 (0.12)	NS	141	317
B cells	0.41 (0.12)	NS	0.55	0.80	0.14 (0.11)	NS	0.15	0.86
monocytes	0.26 (0.11)	0.11 (0.05)*	0.42	1.00	0.26 (0.13)	NS	0.32	0.91
**CD14^+^**	**0.04 (0.09)**	**NS**	**0.08**	**1.90**	**0.48 (0.18)**	**NS**	**1.00**	**1.10**
CD16^+^	0.25 (0.13)	NS	0.16	0.47	0.05 (0.09)	0.09 (0.05)*	0.04	0.83

^1^Traits for which the heritability differed significantly between SPF and non-SPF farms are shown in bold (* p < 0.05). ^2 ^White blood cells expressed as no. cells × 10^6^/mL, PBML sub-sets expressed as proportion of mononuclear leucocytes and antigen density expressed as the number of antibody binding sites per cell (see Materials and Methods).

**Table 5:** Direct heritability (h^2^) estimates for acute phase proteins and average daily gain for SPF and non-SPF pigs^1^

	SPF				Non-SPF			
Trait	direct h^2 ^(s.e.)	Pen effect (s.e.)	Genetic variance × 10^-1^	Residual variance × 10^-1^	direct h^2 ^(s.e.)	Pen effect (s.e.)	Genetic variance × 10^-1^	Residual variance × 10^-1^
								
APP, μg/ml:								
								
haptoglobin	0.23 (0.14)	0.11 (0.05)*	1.10	3.20	0.20 (0.11)	NS	0.84	3.20
TTR,^2^	0.28 (0.22)	NS	0.24	0.36	0.12 (0.18)	NS	0.11	0.51
CRP	0.20 (0.14)	NS	1.40	5.60	0.13 (0.10)	0.11 (0.06)*	0.93	5.30
AGP	0.49 (0.14)	0.08 (0.05)*	0.59	0.52	0.48 (0.14)	NS	0.56	0.51
								
ADG, kg/d	**0.40 (0.07)**	0.09 (0.03)*	**0.07**	**0.09**	**0.13 (0.07)**	**NS**	**0.02**	**0.15**

^1^Traits for which the heritability differed significantly between SPF and non-SPF farms are shown bold (* p < 0.05).

^2^For transthyretin (TTR), both pen and dam effects were fitted

However, the opposite trend was observed for some traits, viz. the proportions of mononuclear and polymorphonuclear cells, CD14^+ ^cells and CD8α^+ ^cells. For these traits, a lower health status environment was associated with higher genetic variance and this difference was significant for CD14^+ ^cells (p < 0.05). Indeed, under SPF conditions, the heritability estimates were not significantly different from zero for the proportions of mononuclear and polymorphonuclear cells and CD14^+ ^cells, but there was a strong pen effect for mononuclear and polymorphonuclear cells within SPF conditions. These two traits will be correlated with each other since the composite of the two traits is equal to one hundred percent. CD4^+ ^and CD4^+^CD8^- ^cells are also somewhat, albeit not significantly, more heritable under SPF conditions (h^2 ^for SPF and non-SPF conditions were 0.78 and 0.57 for CD4^+ ^cells and 0.82 and 0.45 for CD4^+^CD8^- ^cells). Indeed the CD4^+^CD8^- ^subset under SPF conditions had the highest heritability of all. Heritabilities for CD8 antigen density measurements were similar under both SPF and non-SPF conditions (data not shown).

Heritability estimates for the proportion of monocytes and acute phase protein, AGP were unaffected by health status. Further, the health status environment did not affect the maternal effect associated with transthyretin (0.24 (0.11) for SPF conditions, and 0.24 (0.10) for non-SPF conditions).

Heritability estimates for average daily gain were lower under non-SPF conditions compared to SPF conditions (p < 0.01), due to both lower genetic variance and increased residual variance. To explore possible effects of the allocation of different genetic lines to different farms, the data were reanalyzed using only progeny derived from the same sires from sources 2 and 3 (see Table 1), which were equally distributed between SPF and non-SPF farms. Each source was made up of a single genetic line. Within this subset, the same result was observed as for the whole dataset, i.e. heritability estimates for average daily gain were lower under non-SPF than SPF conditions (p < 0.05) (data not shown).

Since the initial weight (start weight) had a significant effect upon some of the traits, e.g. AGP, uni-variate analyses for each trait were repeated with this factor fitted as an extra covariate; however this did not affect the heritability estimates (data not shown).

### Effect of health status on correlations of immune traits with average daily gain

The effect of health status upon the relationship between immune traits and average daily gain is shown in Table 6. Most of the genetic correlations between immune traits and average daily gain that were significantly different from zero were negative, i.e. decreasing average daily gain was associated with increasing values of a particular immune trait. In particular, there were negative genetic correlations between average daily gain and the proportions of CD11R1^+ ^cells, monocytes, the acute phase protein, AGP. There was a strong negative genetic correlation between the proportions of CD11R1^+ ^cells and average daily gain under non-SPF conditions but this relationship was absent under SPF conditions. There were also weak negative phenotypic correlations between these two traits under both SPF and non-SPF conditions. For the proportions of monocytes, there were negative genetic and phenotypic correlations between this trait and average daily gain under SPF conditions but not under non-SPF conditions. There were negative genetic and phenotypic correlations between AGP and average daily gain under both types of environment.

**Table 6:** Correlations between immune traits and average daily gain for SPF and non-SPF pigs^1, 2^

Farm	SPF			Non-SPF		
Trait	r_g_	r_p_	r_e_	r_g_	r_p_	r_e_
WBC	-0.06 (0.24)	-0.03 (0.05)	-0.02 (0.12)	-0.69 (0.36)	-0.10 (0.05)	-0.32 (0.10)
						
MNL%	-0.56 (0.44)	0.09 (0.07)	0.35 (0.16)	-0.32 (0.37)	0.16 (0.06)	-0.11 (0.12)
PMNL%	0.75 (0.40)	-0.07 (0.07)	-0.40 (0.16)	-0.50 (0.34)	**-0.17 (0.06)**	-0.04 (0.13)
						
PBML subsets:						
						
CD4^+^	-0.15 (0.16)	-0.05 (0.05)	0.13 (0.23)	-0.10 (0.33)	-0.02 (0.06)	-0.03 (0.16)
CD8α^+^	-0.35 (0.20)	-0.09 (0.05)	0.10 (0.13)	-0.08 (0.36)	-0.01 (0.07)	-0.06 (0.18)
CD8αβ^+^	0.01 (0.25)	-0.03 (0.06)	-0.05 (0.18)	-0.31 (0.42)	0.04 (0.08)	-0.07 (0.15)
CD11R1^+ ^total	-0.08 (0.20)	**-0.14 (0.05)**	-0.19 (0.13)	**-0.68 (0.29)**	**-0.16 (0.06)**	-0.05 (0.12)
CD11R1^+ ^CD8^+^	-0.25 (0.19)	-0.11 (0.05)	0.01 (0.14)	-0.44 (0.40)	-0.05 (0.07)	-0.10 (0.14)
CD11R1^+ ^CD8^-^	0.20 (0.21)	-0.07 (0.05)	-0.27 (0.13)	-0.34 (0.39)	**-0.18 (0.07)**	-0.12 (0.13)
γδ^+ ^T cell	0.13 (0.19)	**0.17 (0.05)**	0.22 (0.13)	-0.24 (0.39)	**0.15 (0.06)**	-0.28 (0.11)
B cell	-0.01 (0.22)	-0.03 (0.05)	-0.06 (0.13)	-0.33 (0.53)	-0.05 (0.06)	-0.12 (0.10)
monocytes	**-0.46 (0.23)**	**-0.17 (0.05)**	-0.01 (0.12)	-0.27 (0.39)	-0.02 (0.06)	-0.11 (0.11)
CD14^+^	-0.33 (0.68)	**-0.18 (0.06)**	-0.18 (0.13)	-0.44 (0.38)	-0.07 (0.07)	-0.37 (0.19)
CD16^+^	-0.38 (0.33)	**-0.19 (0.07)**	-0.09 (0.17)	-^3^	-^3^	-^3^
						
APP, μg/ml:						
						
haptoglobin	-0.18 (0.34)	**-0.27 (0.06)**	**-0.33 (0.17)**	-0.13 (0.46)	**-0.30 (0.05)**	**-0.34 (0.09)**
TTR,	-0.33 (0.47)	**-0.16 (0.05)**	0.26 (0.16)	-0.80 (0.43)	-0.09 (0.07)	-0.12 (0.14)
CRP	-0.12 (0.41)	-0.10 (0.07)	-0.09 (0.16)	-0.22 (0.54)	-0.05 (0.06)	-0.01 (0.11)
AGP	**-0.53 (0.20)**	**-0.49 (0.05)**	**-0.46 (0.15)**	**-0.72 (0.22)**	**-0.48 (0.04)**	**-0.42 (0.10)**

^1^Correlations significantly different from zero are shown in bold. ^2 ^White blood cells expressed as no. cells × 10^6^/mL, PBML sub-sets expressed as proportion of mononuclear leucocytes and antigen density expressed as the number of antibody binding sites per cell (see Materials and Methods). ^3 ^Did not converge.

Although there were apparently high genetic correlations between other immune traits and weight gain, e.g. WBC and weight gain under non-SPF conditions, these were not significantly different from zero (p > 0.05).

There was a negative phenotypic correlation between haptoglobin and average daily gain, and a weak positive phenotypic correlation between the proportions of γδ^+ ^T cells and average daily gain, with neither of these correlations being affected by health status. There were weak negative correlations between average daily gain and the proportion of PMN leucocytes under non-SPF conditions and between CD14^+ ^cells and average daily gain under SPF conditions only.

The analysis was repeated for each set of traits with the initial weight included as an extra covariate. This extra fixed effect did not affect the genetic or phenotypic relationship between any of the immune traits tested and average daily gain except for the correlations with the proportions of CD11R1^+ ^cells and AGP under non-SPF conditions, and the correlation with the proportions of monocytes under SPF conditions. Adding the initial weight as an extra fixed effect caused the genetic correlation (r_g _(se)) between CD11R1^+ ^cells and average daily gain to increase from -0.68 (0.29) to -0.99 (0.23), and the genetic correlation between AGP and average daily gain to increase from -0.72 (0.22) to -0.92 (0.22). Adding the initial weight as an extra covariate also caused the genetic correlation of average daily gain with the number of monocytes to decrease from -0.46 (0.23) to -0.36 (0.19) and this effect was then no longer significant (0.05 < p < 0.1).

### Correlations between acute phase proteins and PBML subsets

Phenotypic correlations between acute phase proteins and PBML subsets were mostly weak (r < 0.2) and not significantly different from zero (data not shown). However, some genetic correlations were significantly different from zero, and these correlations were all positive. In summary, there was a positive genetic correlation between the concentration of C-reactive protein (CRP) and the proportions of B cells (r_g _= 0.80 (s.e. 0.21)), and between the concentration of haptoglobin and either the proportions of monocytes (r_g _= 0.52 (s.e. 0.24)) or the proportions of CD11R1^+ ^cells (r_g _= 0.53 (s.e. 0.21)).

### Correlations between different PBML subsets

Nearly all genetic and phenotypic correlations between different PBML subsets were not statistically significant from zero except there were strong genetic and phenotypic correlations between pairs of subsets where one subset was part of the other subset e.g. CD4^+ ^and CD4^+^CD8α^+ ^cells (data not shown).

## Discussion

It is essential that markers for increased resistance to infectious disease can be transmitted across generations, i.e. are heritable. Although we have previously estimated the heritability of a range of peripheral blood mononuclear leucocyte subsets and their correlations with performance, we had not yet been able to robustly examine the influence of health status upon these parameters [6]. Our current dataset comprised animals that were previously tested [6] along with additional animals from farms that varied in health status. This data also included additional immune traits such as CD8α^+ ^cell subsets and acute phase proteins, AGP, haptoglobin, CRP and transthyretin.

Overall, most of the immune traits tested were found to be moderately heritable across the dataset as a whole and these heritabilities, combined with the observed trait variability, would permit selection for altered trait values. Estimated heritabilities for total and differential white blood cell (WBC) counts and the acute phase protein, haptoglobin are similar to those quoted by other workers [26] (Diack (unpublished observations)). Our heritability estimates for total and differential white blood cell counts were within the range of those published by Edfors-Lilja *et al*. (1994) and Henryon *et al*. (2006) [26,27]. Additionally, we were able to provide some novel heritability estimates. For example, unlike humans and other species, pigs possess high proportions of CD4^+^CD8α^+ ^cells and we have provided the first evidence that these subsets are heritable. This is arguably unsurprising because these cells are a subset of total CD4^+ ^cells which are also highly heritable, and a high genetic correlation was observed between CD4^+^CD8α^+ ^cells and CD4^+ ^cells.

There was an unexplained maternal effect associated with the acute phase protein, transthyretin which might not necessarily be immune-related. Transthyretin mainly acts as a carrier protein for thyroxine and retinol (vitamin A) [28]. It is also a marker for nutritional status and is usually maintained at high levels except during infection and malnutrition, when it drops [28,29]. Maternal influences such as maternal stress or nutrition have been shown to influence transthyretin levels in the off-spring [30,31], and these effects may well explain our observed maternal effect.

Most immune traits were heritable regardless of health status although some immune traits, e.g. the proportions of mononuclear and polymorphonuclear cells and CD14^+ ^cells, were only observed to be heritable within a lower health status environment. This is possibly because genetic differences are more fully expressed for this particular trait when there are environmental or pathogen challenges. In our experiments, monocytes were all SIRPα^+ ^but only a proportion of them were CD14^+ ^and, unlike for CD14^+ ^cells, health status did not affect the heritability of monocytes. Pig peripheral blood monocytes are a heterogeneous population, both with respect to function and phenotype and, in pigs, CD14^+ ^cells have been suggested to represent a more mature population of monocytes [12,32]. In contrast, other work has shown that exposure to viral or bacterial pathogens can influence the expression of CD14 on porcine alveolar macrophages or dendritic cells [33,34]. Thus, there might be a stronger genetic influence upon either monocyte differentiation or the expression of CD14 in response to the environmental pathogens present within the lower health status environment.

Ideally, markers for increased resistance to infectious disease should correlate (within a herd) with indicators of health, such as performance, morbidity or mortality. Previous work by ourselves and others, has demonstrated negative phenotypic and genetic relationships between some immune traits and weight gain [4,13,14,35]. This study confirms and extends these earlier findings. This type of association could reflect a response to sub-clinical infection that increases the proliferation of certain immune cell types and/or the production of acute phase proteins, with a reduction in growth being a consequence of infection. The traits that were most strongly and consistently associated with weight gain included the proportions of CD11R1^+ ^cells and monocytes and acute phase proteins, AGP and haptoglobin. There were also negative genetic correlations between average daily gain and immune traits, total CD11R1^+ ^cells, monocytes and AGP. For total CD11R1^+ ^cells, this effect was only detectable under non-SPF conditions. The cell marker CD11R1 is mainly expressed by NK cells [5,6] which are one of the major defences against intra-cellular pathogens [36]. Since the main pathogens present on the non-SPF farms were intra-cellular pathogens, e.g. pig circovirus (PCV) and *Mycoplasma hyopneumoniae*, then the genetic association between CD11R1^+ ^cells and weight gain may reflect a response to sub-clinical infection. This effect is reinforced by the observation that correcting for starting weight strengthened the correlation of average daily gain with CD11R1^+ ^cells. This, under this type of non-SPF environment, CD11R1^+ ^cells could act as an indicator for sub-clinical infection.

For monocytes, the genetic relationship between weight gain and this cell type was only evident under SPF conditions. We cannot fully explain this effect. One major difference between the SPF and non-SPF animals was that many of the non-SPF animals came from farms that were positive for PMWS. PCV is one of the main agents associated with PMWS and this virus only appears to infect monocyte/macrophage cell types [37,38]. If viral infection prevented these cell types from proliferating in response to infection, then this could have affected the relationship between these cell types and weight gain under non-SPF conditions.

Unlike CD11R1^+ ^cells, health status did not affect the genetic and phenotypic relationships between AGP and weight gain, which might indicate that this is a more reliable indicator for selection purposes. As with other APP, infection can increase the production of AGP through cytokines TNF α, IL-1 and IL-6. These cytokines can also reduce growth by inducing anorexia and tissue breakdown [39-41]. Since AGP concentrations remain raised for longer after infection than other acute phase proteins such as CRP and haptoglobin, AGP has been used as a marker of sub-clinical infection in large scale human studies [42,43].

In contrast, an alternative view is the lack of any impact of health status on the relationship between AGP and weight gain might indicate that the association between AGP and weight gain is not due to an underlying response to infection, since AGP is also a constitutive protein. High plasma AGP concentrations are present after birth and gradually decrease with age [44,45]. One argument against this view is that the expression of higher levels of AGP has been associated with pro- and anti-inflammatory effects which can influence the outcome of infection and inflammation [46,47]. In one study, higher constitutive levels of AGP present in transgenic mice were associated with higher levels of weight loss and inflammation in response to inflammatory bowel disease compared to wild-type mice [48]. An analogous situation may exist in pigs where animals with higher constitutive serum AGP concentrations are more susceptible to pro-inflammatory tissue damage due to infection, which may lead to reduced weight gain. This hypothesis could be tested by monitoring AGP and weight gain in response to direct challenge or disease outbreak, or by looking for genes that influence both plasma AGP levels and weight gain.

One limitation of this study was that health status was confounded with farm (i.e. housing and environment), although husbandry methods were similar between farms. We attempted to minimise the impact of this confounding by statistically accounting for farm in our models, by placing little importance on mean trait values, as these could differ for many reasons, but concentrating instead on genetic variation and relationships between variables. Performing larger studies on a single farm would enable us to select genetic parameters that could be applied to specific health status situations. However, the generality of our results would be reduced, as we wish to find parameters that are robust across a wide range of health environments.

## Conclusion

Overall, we have shown that for a wide range of immune traits, heritabilities were generally unaffected by health status, although genetic correlations between performance and CD11R1^+ ^cells or monocytes, were influenced by health status. There were strong genetic and phenotypic correlations between AGP and performance, and health status did not affect the strength of these relationships, however the genetic association between CD11R1^+ ^cells and average daily gain was only present under lower health status conditions. In order to effectively select for higher performing animals using either of these measurements, we need to fully understand the underlying mechanisms that control the relationship between these traits and weight gain. Also, the relationship of these immune traits with other immune traits needs to be fully understood to avoid any antagonistic effects. For CD11R1^+ ^cells, we also need to know the genetic correlations between different health status environments. Future use of these biomarkers may be conditional on further studies addressing the implications for complex immune traits of selecting on single markers. In this context, future work should focus on finding genetic markers that are linked to both innate and adaptive immunity and performance, since such markers would be independent of changes in health status and they would avoid logistical issues associated with measurement of phenotypes.

## Abbreviations

Ag: antigen; AGP: alpha-_1 _acid glycoprotein; APP: acute phase protein; CRP: C-reactive protein; EDTA: ethylenediamine tetraacetic acid; ELISA: enzyme-linked immunosorbent assay; IL-1: interleukin-1; IL-6: interleukin-6; LR: Landrace; LW: Large White; MNL:mononuclear leucocytes; NK: natural killer; non-SPF: non specific pathogen-free; SPF: specific pathogen-free; PBML subsets: peripheral blood mononuclear leucocyte subsets; PCV: pig circovirus; PMNL: polymorphonuclear leucocytes; PMWS: porcine multi-wasting syndrome; SIRPα: signal regulatory protein α; TNFα: tumour necrosis factor alpha; TTR: transthyretin; PRRS: porcine reproductive and respiratory syndrome; WBC: white blood cell

## Competing interests

The authors declare that they have no competing interests.

## Authors' contributions

Immune trait assays were set up and performed by MC except for APP assays, haptoglobin, C-reactive protein and transthyretin which were set up and managed by ABD. The data analysis was performed by MC with guidance from OM and SCB. MAM, CG and AH selected the animals used for the study and organized the performance trait measurements and sampling of these animals. This study was conceived by EJG and SCB who were also responsible for obtaining financial support. The manuscript was drafted by MC although all authors have contributed to, read and approved the manuscript.
